# Neuregulin repellent signaling via ErbB4 restricts GABAergic interneurons to migratory paths from ganglionic eminence to cortical destinations

**DOI:** 10.1186/1749-8104-7-10

**Published:** 2012-02-29

**Authors:** Hao Li, Shen-Ju Chou, Tadashi Hamasaki, Carlos G Perez-Garcia, Dennis DM O'Leary

**Affiliations:** 1 Molecular Neurobiology Laboratory, The Salk Institute for Biological Studies, 10010 N. Torrey Pines Rd, La Jolla, CA 92037, USA; 2 Current address: Groth & Co. KB, Birger Jarlsgatan 57B, PO Box 6107, 102 32 Stockholm, Sweden; 3 Current address: Institute of Cellular and Organismic Biology, Academia Sinica, 128 Academia Road, Sec 2, Nangang, Taipei, 115, Taiwan; 4 Current address: Department of Neurosurgery, Kumamoto University, 1-1-1 Honjo, Kumamoto 860-8556, Japan

## Abstract

**Background:**

Cortical GABAergic interneurons (INs) are generated in the medial ganglionic eminence (MGE) and migrate tangentially into cortex. Because most, if not all, migrating MGE-derived INs express the neuregulin (NRG) receptor, ErbB4, we investigated influences of Nrg1 isoforms and Nrg3 on IN migration through ventral telencephalon (vTel) and within cortex.

**Results:**

During IN migration, NRG expression domains and distributions of ErbB4-expressing, MGE-derived INs are complementary with minimal overlap, both in vTel and cortex. In wild-type mice, within fields of NRG expression, these INs are focused at positions of low or absent NRG expression. However, in ErbB4-/- HER4^heart ^mutant mice in which INs lack ErbB4, these complementary patterns are degraded with considerable overlap evident between IN distribution and NRG expression domains. These findings suggest that NRGs are repellents for migrating ErbB4-expressing INs, a function supported by *in vitro *and *in vivo *experiments. First, in collagen co-cultures, MGE-derived cells preferentially migrate away from a source of secreted NRGs. Second, cells migrating from wild-type MGE explants on living forebrain slices from wild-type embryonic mice tend to avoid endogenous NRG expression domains, whereas this avoidance behavior is not exhibited by ErbB4-deficient cells migrating from MGE explants and instead they have a radial pattern with a more uniform distribution. Third, ectopic NRG expression in the IN migration pathway produced by *in utero *electroporation blocks IN migration and results in cortex distal to the blockade being largely devoid of INs. Finally, fewer INs reach cortex in ErbB4 mutants, indicating that NRG-ErbB4 signaling is required for directing IN migration from the MGE to cortex.

**Conclusions:**

Our results show that NRGs act as repellents for migrating ErbB4-expressing, MGE-derived GABAergic INs and that the patterned expression of NRGs funnels INs as they migrate from the MGE to their cortical destinations.

## Background

The ventricular zone (VZ) of dorsal telencephalon (dTel) is the origin of excitatory glutamatergic pyramidal neurons, which migrate radially and establish the six-layered laminar pattern characteristic of mammalian neocortex. In contrast, inhibitory cortical interneurons (INs), which use γ-amino butyric acid (GABA) as their main neurotransmitter, are primarily generated in the ganglionic eminence (GE) of ventral telencephalon (vTel) and enter the cerebral cortex by tangential migration [1].

The GE is subdivided into three components: the lateral ganglionic eminence (LGE), the medial ganglionic eminence (MGE) and the caudal ganglionic eminence (CGE). The LGE mainly generates GABAergic and dopaminergic INs destined to the olfactory bulb, as well as striatal projection neurons [2]. The MGE and CGE give rise to most of the GABAergic INs that migrate tangentially into dTel, including neocortex and hippocampus [2-7]. MGE-derived GABAergic INs migrate through the cortex within the marginal zone (MZ) and the interface between the intermediate zone (IZ) and the subventricular zone (SVZ). After these INs reach their appropriate cortical location, they migrate to their final cortical layer by using either multimodal migration [8] or radial migration perpendicular to their original tangential paths in the MZ and IZ [9].

The major tangential migratory routes of the telencephalic GABAergic INs have been described [10,11], but the mechanisms regulating the migration of INs are not fully understood. Chemorepulsive factors, such as semaphorins, produced by the vTel and chemoattractive activities, such as hepatocyte growth factor, brain-derived neurotrophic factor/neurotrophin-4, glial cell-line derived neurotrophic factor and cytokine Cxcl12/SDF-1, produced by the dTel are reported to influence the tangential migration of INs from vTel to dTel [12-19].

Neuregulin (NRG) signaling plays essential roles in neuronal migration during the development of the vertebrate central nervous system and in the adult forebrain [20-23]. Of the four known members of NRGs (Nrg1 to Nrg4), only Nrg1 and Nrg3 are expressed in the brain during embryonic development [24]. Nrg1, which is the most characterized and complex, has three isoforms as a result of different promoter usages and variant RNA splicing. Nrg1-type I and -type II are also referred to as Nrg1-Ig because of their extracellular Ig-like domain (Ig) while Nrg1-type III is also known as Nrg1-CRD because of a transmembrane cystein rich domain (CRD) [21,24]. Functional activity of these NRGs has been assigned to their unique extracellular epidermal growth factor (EGF)-like domains, which are released as a diffusible form by proteolytic cleavage. These EGF-like domains are necessary and sufficient for the biological activities of NRGs by binding to the ErbB family of tyrosine kinase receptors, which consists of four members (ErbB1 to ErbB4) that form homo- or heterodimers to be functionally active [20,21,25-27].

Most, if not all, GABAergic INs that migrate from the MGE to the cerebral cortex express ErbB4, and a subpopulation of the GABAergic INs expresses ErbB1/EGF receptor (EGFR); ErbB2 and ErbB3 are mostly absent from these migrating INs [28,29]. Nrg1 isoforms have been reported to be expressed coincident with the subpallial migratory path of ErbB4-expressing INs derived from the MGE, and to act as attractants to guide these INs into the cerebral cortex [23]. In the present study, we have also investigated the role of NRG signaling in controlling the migration of MGE-derived GABAergic INs to the cortex. However, in contrast to the report that NRGs are coincident with migrating ErbB4-expressing INs [23], we find that the NRGs are expressed in patterns that outline the paths of the migrating ErbB4-expressing INs, suggesting that the ErbB4-expressing INs avoid the NRG expression domains. Consistent with this suggestion, we show that these complementary patterns of NRG expression and the distribution of ErbB4-expressing INs observed during normal development are substantially degraded in ErbB4-/- HER4^heart ^mutant mice [30] that have a targeted deletion of ErbB4 but are viable because of selective expression of an ErbB4 transgene that rescues early lethality due to defects in heart development.

We interpret these findings to indicate that the NRGs act as repellents or inhibitors of the migration of MGE-derived INs, a function that differs from that previously reported [23]. In support of our conclusion, we present evidence from *in vitro *cell migration assays that the NRGs repel and inhibit the migration of MGE cells. Further, we show *in vivo *using *in utero *electroporation that ErbB4-expressing, MGE-derived INs avoid ectopic domains of NRG expression in their subpallial migration pathway and that positions of cortex distal to the migration block are deficient for ErbB4-expressing INs. In summary, we find that the Nrg1 isoforms are chemorepellents and inhibitors for MGE-derived, GABAergic INs, and that NRG repellent signaling via ErbB4 helps to define the migratory paths of GABAergic INs and acts to funnel them through the forebrain to their cortical destinations.

## Results

### Distributions of ErbB4-expressing interneurons migrating from MGE to cortex show minimum overlap with expression domains of Nrg1 isoforms and Nrg3

**Figure 1:**
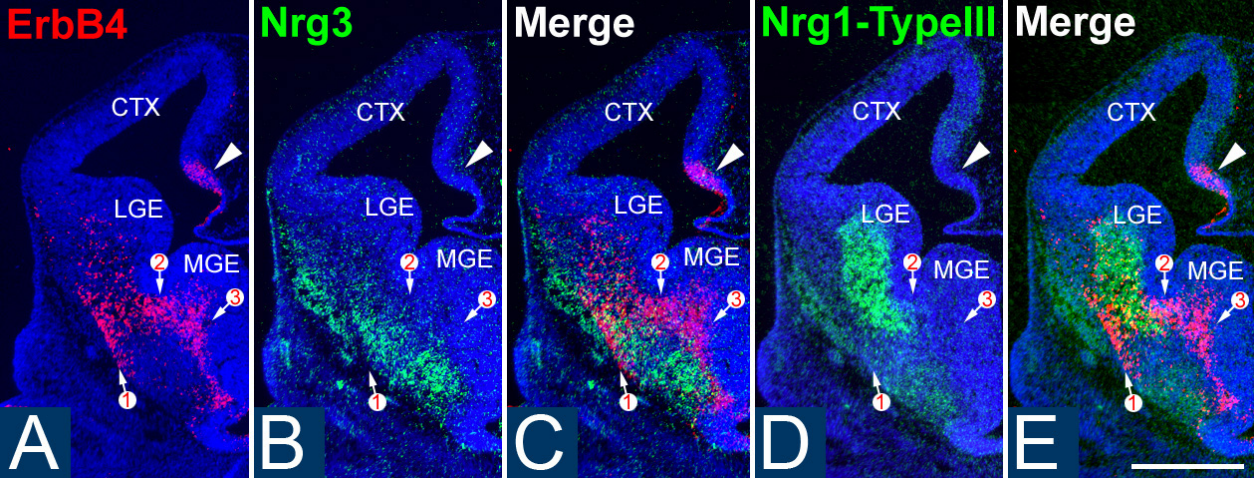
**Patterns of NRG expression and distribution of ErbB4-expressing INs are complementary at E12.5**. **(A-E) ***In situ *hybridization on coronal sections through E12.5 mouse forebrain for expression of ErbB4 (A), marking primarily INs generated in the MGE and migrating to the cortex, and the neuregulins Nrg3 (B) and the type III isoform of Nrg1 (D); (C, E) merged images shown in (A, B), and (A, D), respectively. (A) ErbB4-expressing cells are detected in the ventral telencephalon and cortical hem (arrowhead). Nrg3 (B) and Nrg1-type III (D) are robustly expressed in the mantle zone of the vTel. Most of the ErbB4-expressing INs within the vTel are found at locations with low or undetectable levels of Nrg3 and Nrg1-type III expression, as shown on merged adjacent sections (C, E). Numbered arrows indicate the different migrating streams of ErbB4-expressing cells at matching sites on all sections. CTX, cerebral cortex; LGE, lateral ganglionic eminence; MGE, medial ganglionic eminence. Scale bar: 500 μm.

**Figure 2:**
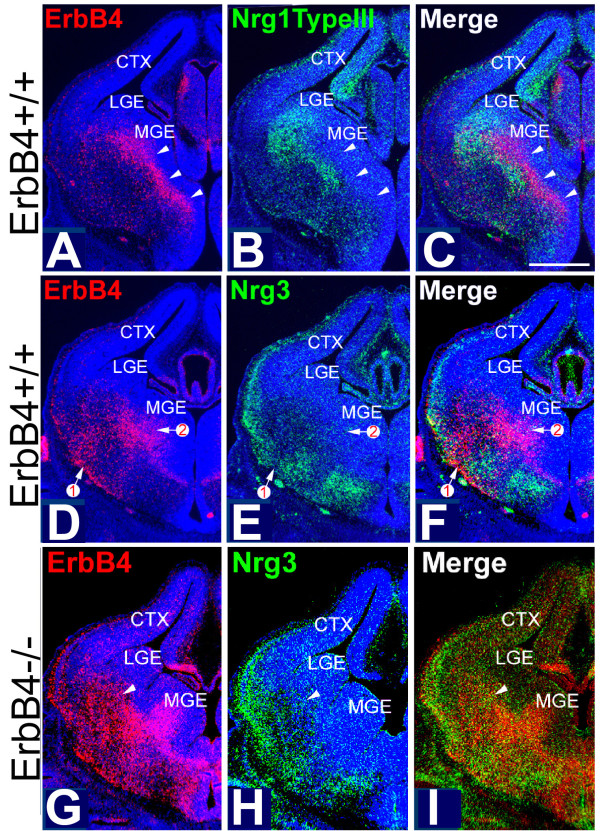
**Complementary patterns of NRG expression and distribution of ErbB4-expressing INs at E13.5 in WT mice are degraded in ErbB4-deficient mice**. **(A-F) ***In situ *hybridization on coronal sections at two different levels ((A-C) more posterior; (D-F) more anterior) as in Figure 1 but at E13.5; (C, F) are merged images shown in (A, B), and (D, E), respectively. In WT, migrating, ErbB4-expressing, MGE-derived INs (A, D) are distributed in the vTel, and a small number in the MZ and IZ in cortex. Nrg1-type III (B) and Nrg3 (E) are both expressed in the nascent cortical plate and in a largely complementary pattern in the vTel. Most ErbB4-expressing INs are found at locations with low or undetectable levels of expression of Nrg1-type III (C) and Nrg3 (F). Arrowheads in (A-C) and numbered arrows in (D-F) mark the positions of migrating streams of ErbB4-expressing INs at matching sites on adjacent sections. **(G-I) ***In situ *hybridization on coronal sections through forebrain of E13.5 ErbB4-/- HER4^heart ^mice for expression of ErbB4, marking primarily INs generated in the MGE and migrating to the cortex, and Nrg3; (I) merged images shown in (G, H). Compared to their WT littermates (D-F), the distributions of the migrating ErbB4-expressing, MGE-derived INs in the ErbB4 mutants is broader and more diffuse, exhibiting abnormally extensive overlap with the expression domains of Nrg3 (I). Arrowheads mark matching sites on the pair of sections (G-I). CTX, cerebral cortex; LGE, lateral ganglionic eminence; MGE, medial ganglionic eminence. Scale bar: 500 μm.

**Figure 3:**
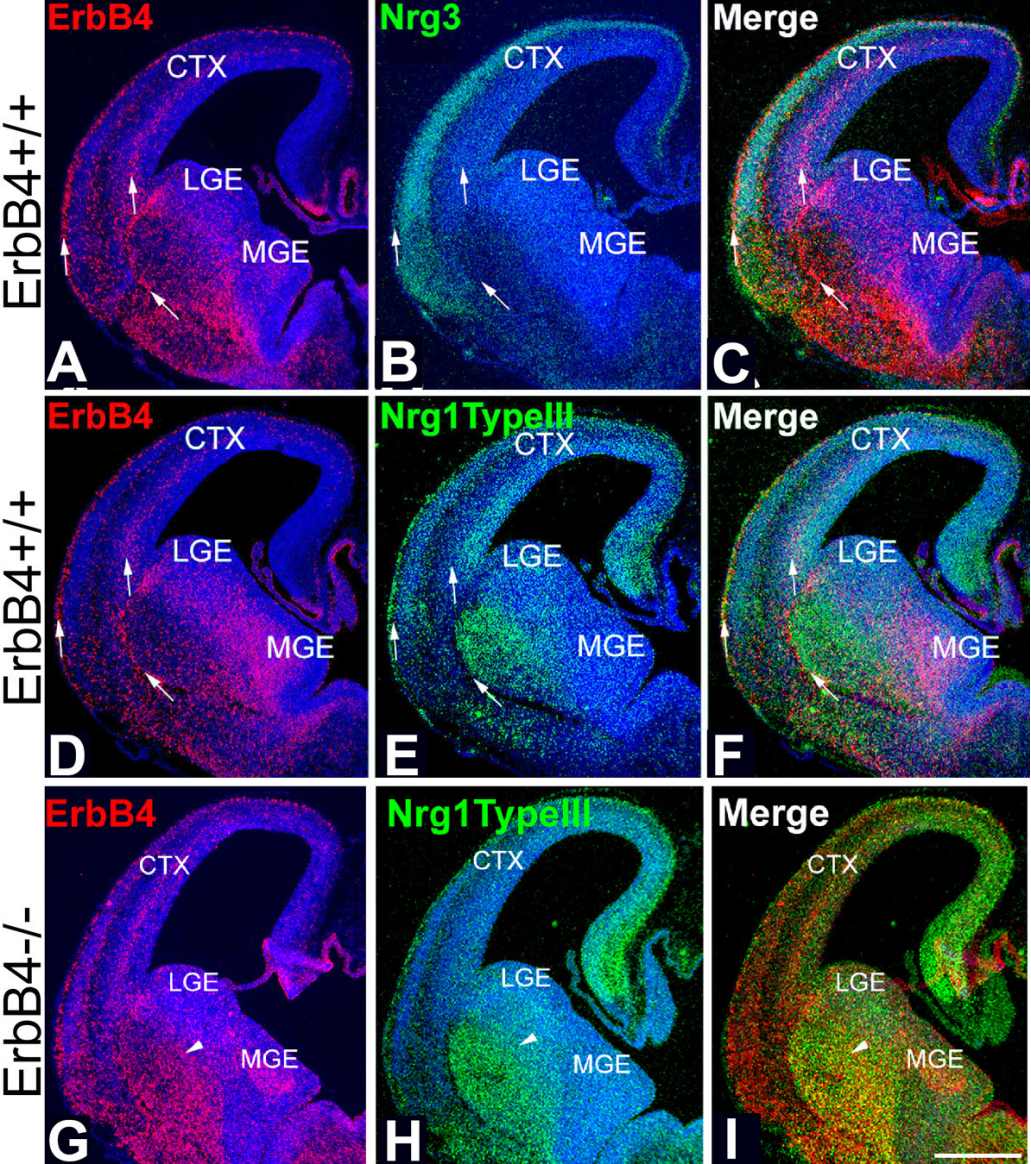
**Complementary patterns of NRG expression and distribution of ErbB4-expressing INs at E14.5 in WT mice are degraded in ErbB4-deficient mice**. **(A-F) ***In situ *hybridization on coronal sections at two different levels ((A-C) anterior; (D-F) more posterior) as in Figure 1 but at E14.5; (C, F) merged images shown in (A, B), and (D, E), respectively. (A, D) In WT, migrating, ErbB4-expressing INs derived from the MGE are detected in the vTel and in their tangential migratory streams in cortex in the marginal zone (MZ) and intermediate zone (IZ) (arrows). (B) Nrg3 is expressed robustly in the cortical plate. (E) Nrg1-type III is highly expressed in the mantle zone of the vTel and in the VZ/SVZ in the dorsal telencephalon. (C, F) ErbB4-expressing INs are preferentially distributed within domains with low or undetectable levels of NRG expression. **(G-I)***In situ *hybridization on coronal sections through forebrain of E14.5 ErbB4-/- HER4^heart ^mice for expression of ErbB4, marking primarily INs generated in the MGE and migrating to the cortex, and the neuregulin Nrg1-type III; (I) merged images shown in (G, H). Compared to their WT littermates (D-F), the distributions of the migrating ErbB4-expressing, MGE-derived INs is much broader and diffuse, exhibiting abnormally extensive overlap with the expression domains of Nrg1-type III (G-I). Arrowheads mark matching sites on the pair of sections (G-I). CTX, cerebral cortex; LGE, lateral ganglionic eminence; MGE, medial ganglionic eminence. Scale bar: 500 μm.

**Figure 4:**
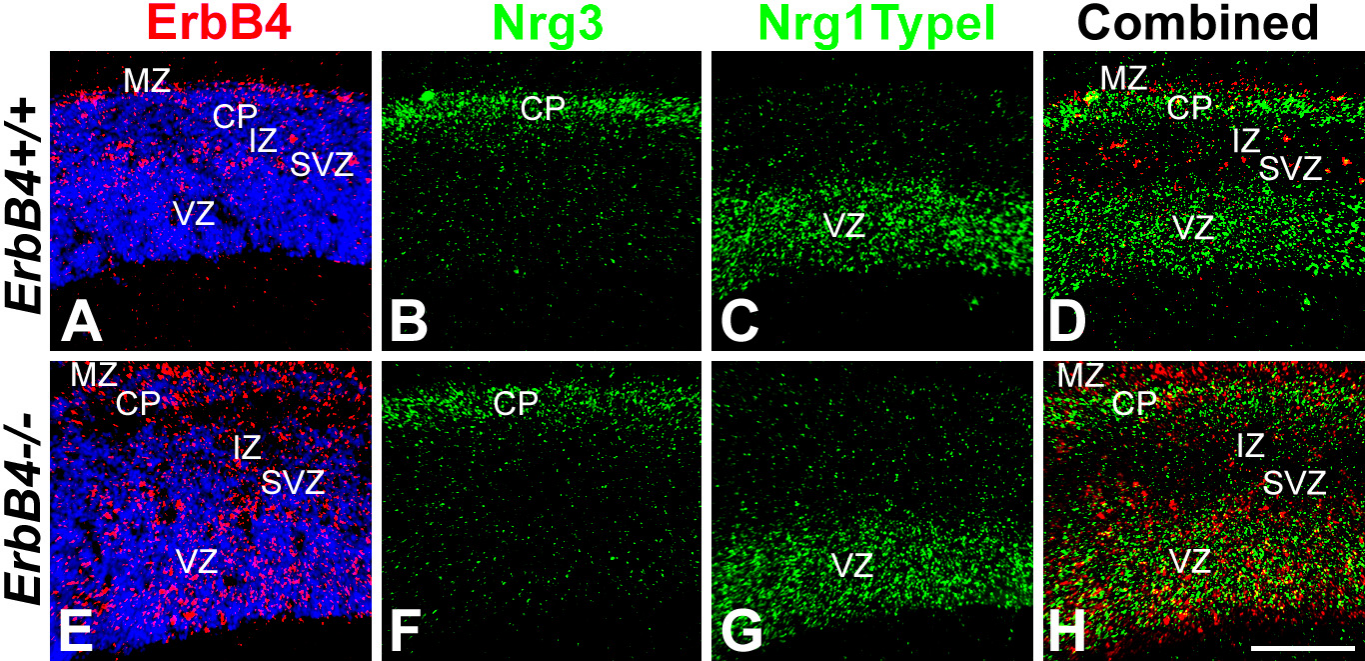
**Patterns of NRG expression and distribution of ErbB4-expressing INs are complementary within neocortex of WT mice and are degraded in ErbB4-deficient mice**. *In situ *hybridization on coronal sections through the cortical wall of littermates of E14.5 WT and ErbB4-/- HER4^heart ^mice for expression of ErbB4, marking INs generated in the MGE and migrating within the marginal zone (MZ) and intermediate zone (IZ) of the cortex, and the neuregulins Nrg3 and the type I isoform of Nrg1. **(A-D) **In WT neocortex, ErbB4 expressing, MGE-derived INs are distributed in the MZ and the interface of IZ and subventricular zone (SVZ) (A), whereas Nrg3 is expressed in the cortical plate (CP) (B) and Nrg1-type I is expressed in the ventricular zone (VZ) and SVZ (C). The MGE-derived INs are largely found outside of the expression domains of Nrg1-type I and Nrg3 (D). **(E-H) **In the neocortex of ErbB4-/- HER4^heart ^mice, MGE-derived INs are more broadly distributed than in WT, with a larger proportion found in the expression domains of Nrg1-type I in the VZ/SVZ and Nrg3 in the MZ (H). Scale bar: 200 μm.

Most cortical GABAergic INs originating from the MGE express the receptor tyrosine kinase ErbB4 [29], which binds NRGs and activates their signaling pathways. Therefore, we used ErbB4 as a marker for these INs to study the relationship between their migratory pathways and the expression patterns of NRGs in the telencephalon in both wild-type (WT) and ErbB4 mutant mice. We first determined the expression pattern of NRGs expressed in embryonic telencephalon during the period of IN migration from the MGE to the cortex, including Nrg1-type I (Nrg1-Ig) and Nrg1-type III (Nrg1-CRD) and Nrg3. In the vTel, the expression of Nrg1-type III and Nrg3 is most prominent. At embryonic day (E)12.5, Nrg3 and Nrg1-type III are robustly expressed within the mantle zone of the vTel, outside of the LGE and MGE, in largely complementary patterns, with the expression of Nrg3 being more lateral and ventral to that of Nrg1-type III (Figure 1). These complementary expression patterns persist at E13.5 (Figure 2) and E14.5 (Figure 3). In the cortex, Nrg1-type I and Nrg1-type III expression is mostly restricted to the VZ/SVZ (Figures 2B and 3E for Nrg1-type III, Figure 4C for Nrg1-type I). In contrast, Nrg3 is predominantly expressed in the forming cortical plate (CP) (Figures 2E, 3B, and 4B).

At each age analyzed, which together encompass the migratory period of GABAergic INs from the MGE to cortex, the great majority of the ErbB4-expressing INs within the vTel are found at locations with low or undetectable levels of Nrg1/Nrg3 expression, although a very small portion of ErbB4-expressing INs do overlap with NRG expression domains (Figures 1C, E, 2C, F, and 3C, F). This complementary relationship between NRGs and MGE INs is also observed within the cortex. There are two main migratory pathways for GABAergic cortical INs: the interface of IZ/SVZ and the MZ (Figure 4A). Interestingly, Nrg1 is expressed in the SVZ/VZ just beneath the IZ/SVZ pathway (Figure 4C), whereas Nrg3 is expressed in the CP just beneath the MZ (Figure 4B). In WT cortex, ErbB4 and NRGs are predominantly expressed in different compartments with minimal overlap (Figure 4A-D). The distribution of ErbB4-expressing INs at these ages is strongly focused on the domains of low NRG expression in the IZ and the MZ, and only a small proportion of ErbB4-expressing INs is found within the NRG expression domains in the CP and the VZ (Figures 3A-F and 4).

These strongly complementary relationships between the NRG and ErbB4 expression patterns suggest that NRGs may play important roles during the tangential migration of the ErbB4-expressing cortical INs. Further, if the NRGs have such a role, the mostly non-overlapping patterns of expression of Nrg1/Nrg3 and ErbB4 indicate that NRGs function as inhibitory or repellent guidance cues for migrating ErbB4-expressing INs, and are inconsistent with NRGs acting as attractants for ErbB4-expressing INs.

### NRGs repel migrating MGE cells *in vitro*

To address the influence of Nrg1 and Nrg3 on the migration of MGE-derived INs, and specifically whether they have a repellent/inhibitory influence or, conversely, an attractant effect, we first used the *in vitro *collagen gel co-culture assay for detecting the effects of secreted molecules such as the NRGs on cell migration. Specifically, we used this co-culture assay to determine the responses of the migrating MGE-derived cells to secreted NRGs. MGE explants isolated from E14.5 mice constitutively expressing enhanced green fluorescent protein (eGFP) [31] were co-cultured at a distance from aggregates of 293T cells transfected with an empty vector as a control, or vectors containing the EGF-like domains of two splice variants of Nrg1 (Nrg1-α and Nrg1-β) or Nrg3, each of which binds ErbB receptors and results in their dimerization and autophosphorylation, as well as activation of the NRG-ErbB4 signaling pathway [21,24,26,27].

**Figure 5:**
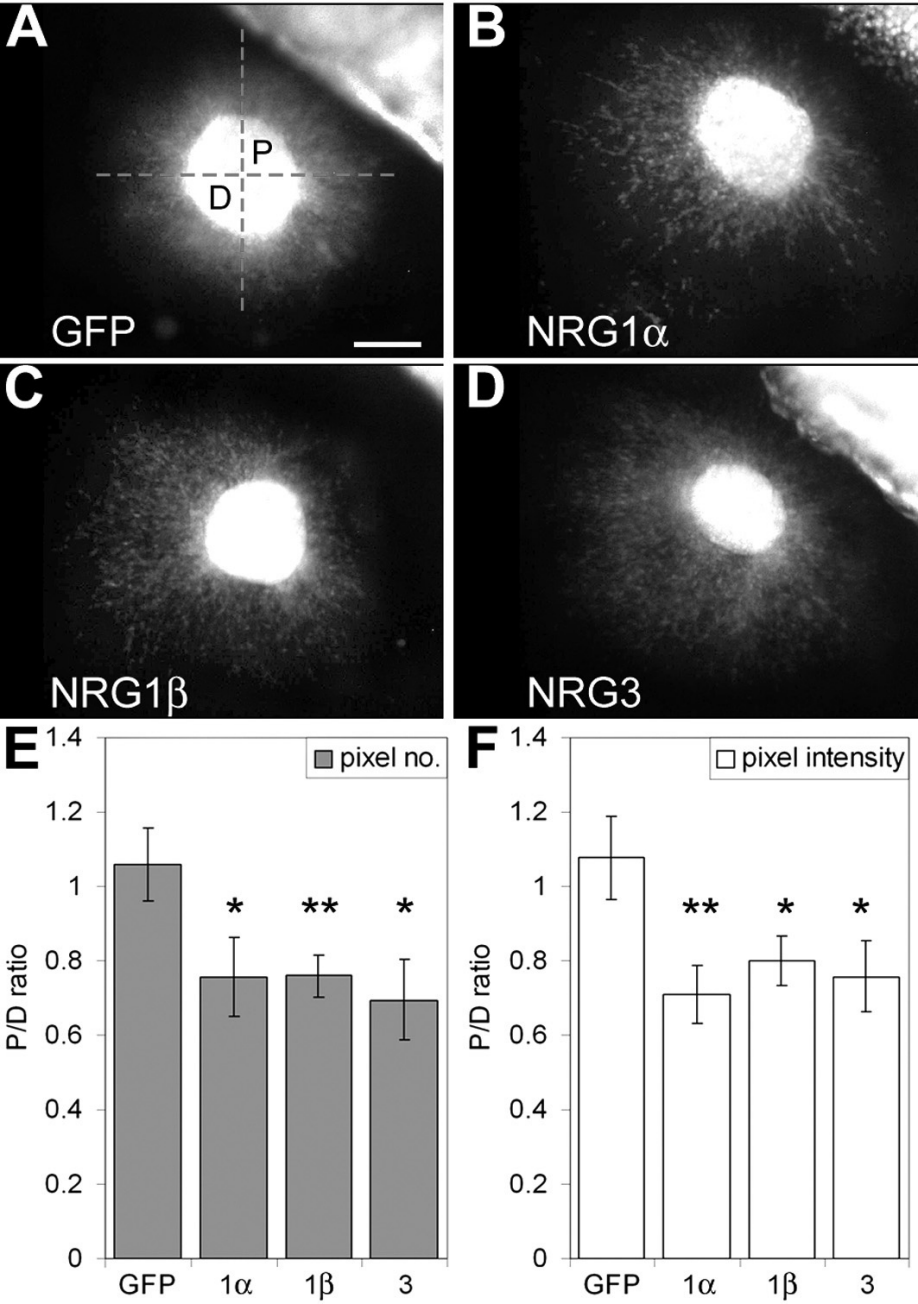
**Secreted NRGs have a repellent effect on cells migrating from MGE explants in collagen co-cultures**. **(A-D) **MGE explants collected at E14.5 from eGFP-expressing mice were co-cultured with aggregates of 293 cells transiently transfected with a control expression construct (A), or a construct expressing the functional Nrg1α EGF-like domain (B), Nrg1β EGF-like domain (C) or Nrg3 EGF-like domain (D). In the control, cells migrate symmetrically from the MGE explant (A). The pattern of cell migration from the MGE explants is asymmetric in the presence of the 293 cell aggregates transfected with the functional NRG EGF-like domains (B-D), with a significant preference for cells to migrate away from the transfected cell aggregates. **(E, F) **Quantification of eGFP fluorescence done blind to the transfection type confirmed the statistical significance of the symmetric versus asymmetric migration. The dashed set of perpendicular lines shown in (A) divide MGE explants into four quadrants. The numbers of cells within the quadrants on the sides of the MGE explants proximal (P) and distal (D) to the transfected cell aggregates were estimated by automated analysis of eGFP fluorescence measuring total pixel number (E) or total pixel intensity (F) as described in Pak *et al. *[48]. Compared to the symmetric pixel measurements in the controls, cells migrating from MGE explants in the presence of the 293 cell aggregates transfected with the functional NRG EGF-like domains exhibit asymmetric pixel measurements, with more pixels in the distal (D) than proximal (P) quadrants (*P < 0.1; **P < 0.01; unpaired Student's *t*-test). Scale bar: 200 μm.

In control co-cultures, cells migrate out of the MGE explants in a symmetric pattern (15 out of 15 explants; Figure 5A). In contrast, in co-cultures with cell aggregates expressing Nrg1 isoforms or Nrg3, the pattern of cell migration from the MGE explants is asymmetric, with 96% of the explants (45 out of 47 explants) exhibiting diminished migration of MGE cells towards the NRG source relative to the robust migration away from it (Figure 5B-D). This qualitative assessment is supported by quantitative analyses that measured the levels of fluorescence of the eGFP reporter that marked cells migrating from the MGE explants. The histograms in Figure 5 depict the data obtained from the most conservative measurement of fluorescence, in which we measured all fluorescence found immediately outside of the explants' borders, including the dense halo of cells abutting the explants that may be formed by cells that move out of the explants' borders passively or prior to coming under the influence of NRG diffusing from the transfected cell aggregates. Nonetheless, these measurements revealed a statistically significant difference in the levels of eGFP fluorescence between the distal (D) and proximal (P) quadrants, indicating significantly fewer cells in the quadrant emanating from the MGE explant facing the NRG source relative to the opposing quadrant (Figures 5E, F). When the measurement are done at positions progressively further away from the MGE explants, and therefore progressively enriching the sample for cells migrating under the influence of the secreted NRGs, the disparity between the quadrant of the MGE explant proximal to the NRG source relative to the opposing quadrant increases dramatically and quickly reaches the point where essentially all of the MGE cells are present in the quadrant distal to the NRG source (equivalent to a P/D ratio of 0 in Figures 5E, F). In contrast, as expected, in control co-cultures the symmetry in the distribution of fluorescence between the proximal and distal quadrants persists at a distance from the MGE explant. These findings demonstrate that, *in vitro*, Nrg1 and Nrg3 have a chemorepellent or inhibitory effect on the migration of MGE cells, consistent with the *in vivo *findings of complementary, non-overlapping distributions of ErbB4-expressing, MGE-derived INs and domains of NRG expression described in the preceding section.

### Complementary patterns in distribution of MGE-derived interneurons and NRG expression domains are degraded in mice deficient for NRG-ErbB4 signaling

In WT mice, we find that as ErbB4-expressing, GABAergic INs migrate from the MGE to cortex, they take paths that have low or undetectable levels of NRG expression and tend to avoid the surrounding domains of NRG expression (Figures 1, 2, 3, and 4). These findings, and our findings from the collagen gel co-culture experiments showing that, *in vitro*, NRGs act as chemorepellents for MGE cells (Figure 5), suggest that most of the ErbB4-expressing, MGE-derived INs avoid NRG expression domains and that NRGs function as inhibitory or repellent guidance cues for them. We have further addressed this issue by analyzing the distributions of ErbB4-expressing, MGE-derived INs relative to NRG expression domains in mice with a targeted deletion of ErbB4, but which express a human ErbB4 transgene under the cardiac-specific α-MHC promoter (HER4^heart^) to rescue the mid-embryonic lethality of the ErbB4 null mutation due to impaired myocardial trabeculation [30]. In these ErbB4-/- HER4^heart ^mice, NRG signaling through ErbB4 is abolished in INs. Therefore, if NRGs have a repellent effect on migrating MGE-derived INs, we predict that the complementary patterns of NRG expression and the distribution of ErbB4-expressing INs observed during normal development would be degraded in ErbB4 mutant mice.

The ErbB4 riboprobe used to determine the distribution of ErbB4-expressing INs in WT mice is directed against a 5' cDNA fragment of mouse *ErbB4 *transcript that persists in the ErbB4-/- HER4^heart ^mice. Therefore, as in WT mice, we were able to use *in situ *hybridization with the ErbB4 riboprobe to visualize MGE-derived INs deficient for ErbB4 in the ErbB4-/- HER4^heart ^mice and directly compare their distributions with the NRG expression patterns. As described in a preceding section (Figures 1, 2, 3, and 4), in WT mice, MGE-derived INs, defined by their expression of ErbB4, exhibit very little overlap with the NRG expression domains, specifically Nrg1-type III and Nrg3. In contrast, ErbB4-expressing, MGE-derived INs are more dispersed in their distribution in ErbB4-/- HER4^heart ^mice (Figures 2G and 3G) compared to their WT littermates (Figures 2A, D and 3A, D), and their distribution overlaps considerably with the expression domains of both Nrg1-type III (Figure 3H, I) and Nrg3 (Figure 2H, I). This degradation in the complementary distributions of ErbB4-expressing INs and NRG expression domains is also observed within the cortex of ErbB4-/- HER4^heart ^mice, as the distribution of ErbB4-expressing INs is more diffuse than in WT and overlaps considerably with the NRG expression domains in the CP and the SVZ/VZ (Figures 2, 3 and 4).

The fact that the patterned distribution of the INs is not completely lost is evidence of the action of other guidance activities that have been reported to affect the migration of MGE-derived INs (see Introduction). Thus, the complementary patterns of NRG expression and the distribution of ErbB4-expressing, MGE derived INs are degraded in ErbB4-/- HER4^heart ^mice, consistent with our *in vitro *findings that NRGs function as inhibitory or repellent guidance cues for ErbB4-expressing, MGE-derived INs.

### Cells migrating from WT MGE but not ErbB4-deficient MGE avoid endogenous WT NRG expression domains

**Figure 6:**
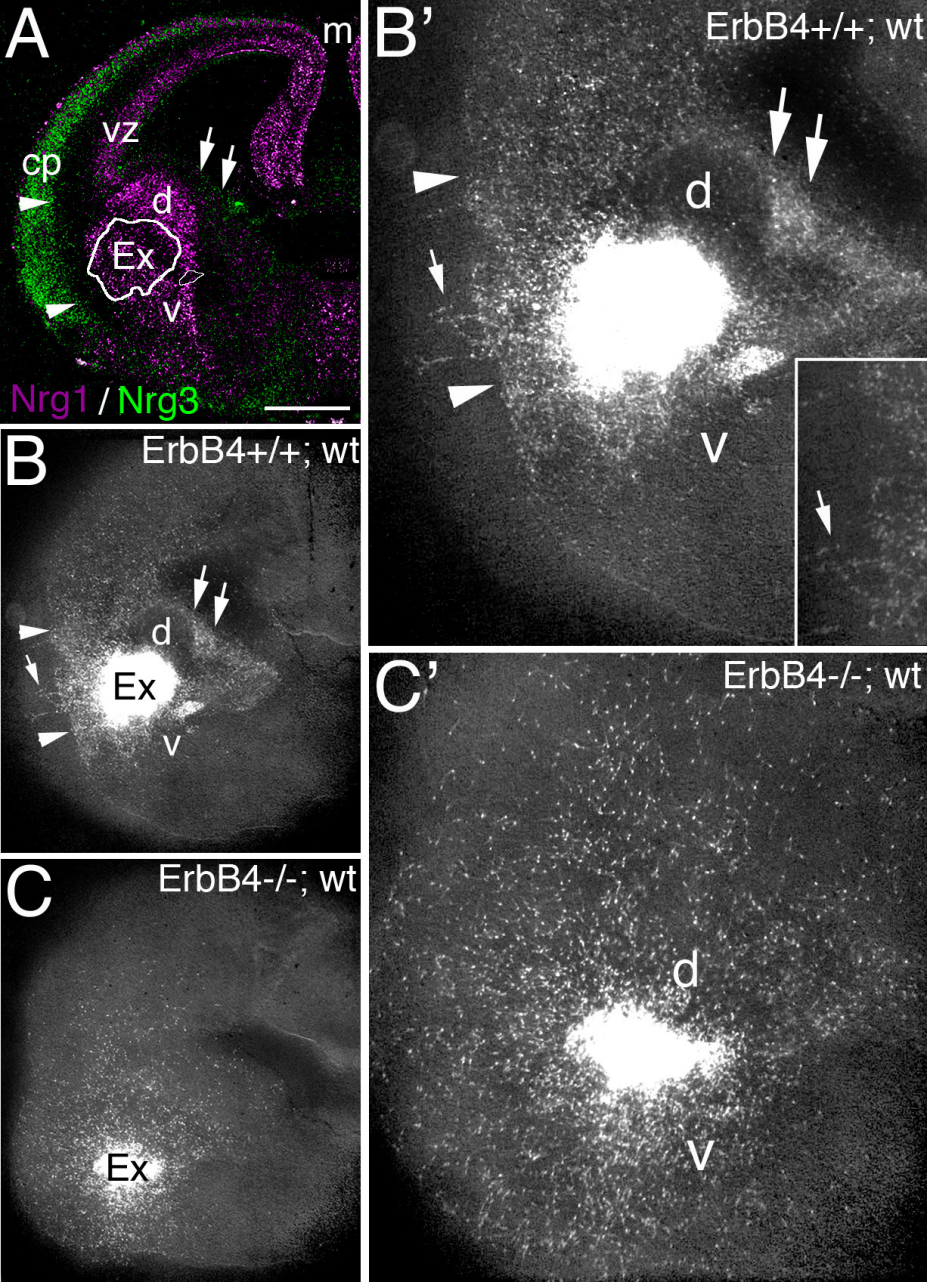
**Cells migrating from MGE explants on live forebrain slices avoid NRG expression domains in an ErbB4-dependent manner**. **(A) **Pseudo-colored merged images of *in situ *hybridizations on coronal sections through E14.5 mouse forebrain for expression of Nrg3 (green) in the cortical plate (cp) and Nrg1-type III (violet) in the mantle zone of the vTel and in the ventricular zone (vz)/SVZ in the dTel. An explant from the medial ganglionic eminence (MGE) from E14.5 WT or ErbB4-/- HER4^heart ^mice crossed to an eGFP reporter line was placed on a living coronal slice through E14.5 WT mouse forebrain, positioned in the mantle zone; the white outline (labeled Ex) marks the explant shown in (B), and approximates explant position in all cases. **(B-C') **MGE explants and cells migrating from them are marked with an eGFP reporter (white label). (B, B') MGE explant from WT eGFP mouse; (B') is higher power. After 48 hours in culture, eGFP-marked cells migrating from MGE explants exhibited little overlap with NRG expression domains, similar to *in vivo *WT (Figures 1, 2, and 3). The migrating cells abut the Nrg3 expression domain in the cp (arrowheads), although a few cells do enter it (arrow in (B, B'); inset in (B')), and also largely avoid domains of robust Nrg1-type III expression; for example, few labeled cells enter the domains dorsal (d) and ventral (v) to the MGE explant. Dual arrows mark the ganglionic eminence abutting the lateral ventricle. (C, C') MGE explant from ErbB4-/- HER4^heart ^eGPF mice; (C') is higher power of (C). Cells deficient for ErbB4 migrating from eGFP-marked MGE explants from E14.5 ErbB4-/- HER4^heart^; eGFP mice, are distributed in a radial pattern distinct from that exhibited by WT MGE explants, and overlapping more with NRG expression domains than do WT cells. Scale bars: 500 μm (A-C); 250 μm (B', C').

To provide additional evidence that INs avoid domains of NRG expression as they migrate from the MGE to the cortex, we compared the distributions of cells that migrate from explants of MGE from E14.5 WT and ErbB4-/- HER4^heart ^mice placed on living coronal slices of E14.5 WT forebrain (Figure 6). The MGE explants and all cells that migrate from them were marked with an eGFP reporter by breeding ErbB4+/- HER4^heart ^mice with mice constitutively expressing eGFP [31]; the distributions of eGFP-marked cells were compared after 48 hours of culture for MGE explants isolated from ErbB4-/- HER4^heart ^eGFP mice or, as a control, ErbB4+/+ HER4^heart ^eGFP mice.

WT cells migrating out of MGE explants from ErbB4+/+ HER4^heart ^eGFP mice are distributed in a pattern that exhibits little overlap with NRG expression domains (n = 4; compare Figure 6B, B' with 6A). These WT eGFP-labeled, MGE-derived cells exhibit patterned distributions with a tendency to avoid domains of high NRG expression in the slices of WT forebrain - for example, the domain of high Nrg3 expression in the CP and the domains of robust Nrg1 expression in the vTel mantle zone. This patterned, complementary distribution is similar to that of ErbB4-expressing INs relative to NRG expression domains in WT mice (Figures 1, 2, and 3). In contrast, on similar living sections of WT forebrain, eGFP-labeled, ErbB4-null cells migrate out of MGE explants from ErbB4-/- HER4^heart ^eGFP mice in a more or less uniform, radial pattern that does not show the preference exhibited by eGFP-labeled, WT MGE-derived cells to avoid NRG expression domains (n = 5; compare Figure 6C, C' with 6A). Thus, in summary, WT MGE-derived cells show a preference to avoid NRG expression domains not seen for ErbB4-null, MGE-derived cells when migrating under the same conditions on living slices of WT forebrain. Further, these findings demonstrate that the avoidance behavior exhibited by WT MGE-derived cells is due to their expression of ErbB4 and provide evidence that the migratory defects of MGE-derived INs in ErbB4-/- HER4^heart ^mice are due to an absence of NRG-ErbB4 signaling autonomous to the MGE-derived INs. These findings support the conclusion that the NRG expression domains in embryonic forebrain inhibit or repel ErbB4-expressing INs as they migrate from the MGE and function during normal development as barriers that funnel migrating INs from the MGE to the cortex.

### Migration of MGE-derived interneurons is blocked *in vivo *by ectopic NRG expression domains and results in a reduction of interneurons in cortex

The evidence that we have obtained from a variety of analyses, including the distributions of ErbB4-expressing INs relative to NRG expression domains in WT mice and mice deficient for NRG-ErbB4 signaling, from *in vitro *co-cultures, and explant migration assays on brain slices, demonstrate that NRGs act as repellents to direct the migration of MGE-derived ErbB4-expressing INs. As an additional assessment of the function of NRGs in influencing the migration of MGE-derived ErbB4-expressing INs, we carried out *in utero *electroporations to produce focal ectopic domains of NRG expression targeted for the migratory path of MGE-derived INs. We predicted that if NRGs are repellents for migrating MGE-derived INs, the INs would avoid a domain of NRG expression ectopically positioned within their migration path, and their migration should be at least partially blocked. On the other hand, if NRGs are attractants for ErbB4-expressing INs, we would predict that MGE-derived INs would not avoid ectopic NRG expression domains and would either accumulate within them and/or would pass through them, similar to the published results of electroporation of the cytokine Cxcl12, a putative chemoattractant for migrating INs [18,32].

**Figure 7:**
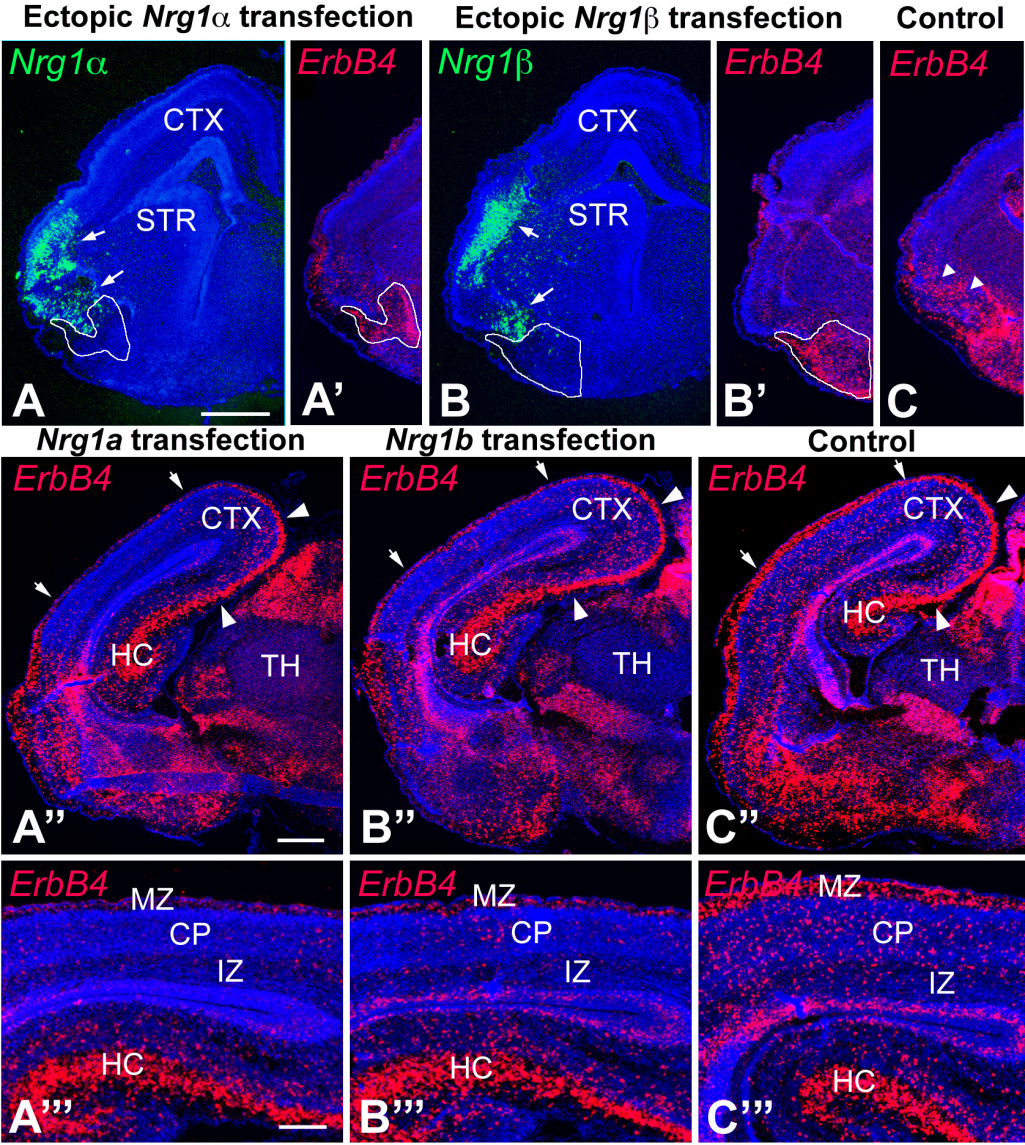
**Tangential migration of ErbB4-expressing INs from MGE to cortex is blocked by NRGs ectopically expressed within their subpallial migratory pathway**. **(A-C) ***In utero *electroporation was performed at E12.5 to ectopically express the functional NRG constructs Nrg1α-EGF (A) and Nrg1β-EGF (B) in the ventral lateral forebrain. At E17.5, ectopic NRG expression domains were visualized by *in situ *hybridization with probes for Nrg1α (A, in green) and Nrg1β (B, in green) and MGE-derived INs were visualized by *in situ *hybridizations with ErbB4 probe (A', B', C, in red) on coronal sections through the transfected brains. In Nrg1-transfected brains, ErbB4-expressing INs do not enter the ectopic Nrg1 expression domain (arrows in (A, B)) and accumulate ventral to it (domains outlined by white line in (A, A', B, B')). In the control brains with similar transfected domains where only an eGFP expression vector was transfected, the ErbB4-expressing INs migrate through the transfected domain (arrowheads in (C)). Within the cortex posterior to the transfected domains, the number of ErbB4-expressing cells is drastically decreased in Nrg1-transfected cerebral cortex (A'', A''', B'', B''')" compared to control (C'', C'''). Arrowheads in (A'', B'', C") shown that the migration of ErbB4-expressing INs derived from the caudal ganglionic eminence that populate the hippocampus and caudomedial cortex is not affected by transfections into the migrational paths of INs from the MGE. (A''', B''', C''') Higher magnification images of the region indicated with arrows in (A'', B'', C''). CP, cortical plate; CTX, cerebral cortex; HC, hippocampus; IZ, intermediate zone; MZ, marginal zone; STR, striatum; TH, thalamus. Scale bars: 1 mm (A, A', B, B', C); 1 mm (A'', B'', C''); 0.5 mm (A''', B''', C''').

*In utero *electroporations of a CAG expression vector containing the EGF domains of mouse Nrg1α, Nrg1β or Nrg3, and eGFP were targeted to the vTel migratory path of MGE-derived INs at E12.5, and the distributions of ErbB4-expressing INs relative to the ectopic NRG expression domains were assessed at E17.5. The CAG promoter drives robust expression in essentially all cell types [33] and resulted in a strong focal expression of NRGs at ectopic sites in the electroporated brains. Ectopic expression domains were initially assessed by visualizing eGFP, and subsequently confirmed using *in situ *hybridization for the relevant NRG; the distribution of MGE-derived INs was assessed by their expression of ErbB4 (Figure 7). We performed Nissl staining on sections of the Nrg1α, Nrg1β or Nrg3, and/or eGFP electroporated brains and did not observe any significant cytoarchitectural changes in these brains (data not shown).

We find that ectopic expression domains of NRGs positioned in the migratory path of MGE-derived INs ventrolaterally within the telencephalon blocked the migration of ErbB4-expressing INs (Figure 7). The majority of ErbB4-expressing INs accumulates at a position in their migratory path proximal to the ectopic expression domain of Nrg1-α (Figure 7A-A'''; n = 4), Nrg1-β (Figure 7B-B'''; n = 4), or Nrg3 (data not shown; n = 3) and relatively few enter it. In contrast, ErbB4-expressing INs migrate into and through the electroporation domain in the control cases electroporated with CAG-eGFP alone (Figure 7C-C'''; n = 5). The distribution of the ErbB4-expressing INs is reminiscent of the distribution of cells migrating from WT MGE explants on living forebrain slices, with both exhibiting a tendency to avoid entering the NRG expression domain, whether the ectopic NRG expression for the *in vivo *electroporation experiments presented here (Figure 7) or the endogenous NRG expression as for the WT MGE explant-forebrain slice migration experiments presented in the preceding section (Figure 6).

These *in vivo *electroporation experiments indicate that NRGs are repellents for migrating MGE-derived INs and that the ectopic domains of NRGs block their migration to the cortex. Consistent with this interpretation, we find that the cortex distal to the ectopic NRG expression domain is virtually devoid of ErbB4-expressing INs (Figure 7A'', A''', B'', B'''). This 'shadow effect' indicates that the ectopic NRG expression domain indeed blocked the migration of MGE-derived, ErbB4-expressing INs, resulting in not only their aberrant accumulation within their subpallial pathway but also in their failure to reach the cortex distal to the NRG blockade. In contrast, the control transfections have no detectable effect on the migration of MGE-derived, ErbB4-expressing INs along either their subpallial path or their distribution in the cortex distal to the control transfection domain (Figure 7C'', C'''). As expected, the migration of ErbB4-expressing INs into caudomedial cortex and the hippocampus is largely unaffected even in the brains electroporated with NRG expression constructs (Figure 7A'', A''', B'', B'''), consistent with their origin in the CGE and their caudal migratory path, which is distinct from the path of MGE-derived INs blocked by the ectopic NRG expression domain [5,7]. In summary, the most straightforward interpretation of these findings is that Nrg1 and Nrg3 inhibit or repel INs migrating from the MGE to the cortex *in vivo*.

### Cortex of ErbB4-/- HER4^heart ^mice has reduced numbers of interneurons

**Figure 8:**
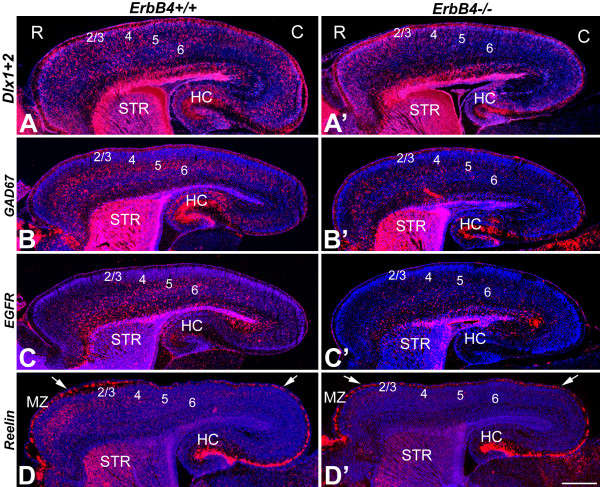
**Cortex of ErbB4-deficient mice has diminished numbers of INs**. **(A-D') ***In situ *hybridizations were performed on sagittal sections of P0 ErbB4+/+ (A-D) and ErbB4-/- HER4^heart ^(A'-D') brains with interneuronal markers, Dlx1/2 (A, A'), GAD67 (B, B'), EGFR (C, C') and Reelin (D, D'). Arrows in (D, D') mark rostral (anterior; left) and caudal (posterior; right) positions in the marginal zone (MZ). Compared to WT (A-D), the number of INs is dramatically decreased in the cortex of the ErbB4-deficient mice (A'-D'). 2/3, layer 2/3; 4, layer 4; 5, layer 5; 6, layer 6; C, caudal; HC, hippocampus; R, rostral; STR, striatum. Scale bar: 500 μm.

Using *in situ *hybridizations with an array of markers for GABAergic INs, including Dlx1/2 [3], GAD67 [34], EGFR/ ErbB1 [35] and Reelin [36], we studied at postnatal day (P)0 the cortical distribution of INs in ErbB4-/- HER4^heart ^mice (Figure 8). Each marker indicated a considerable decrease in the number of INs in the ErbB4-/- HER4^heart ^mice relative to WT littermates (Figure 8). Further, each marker showed that the density of INs in the ErbB4-/- HER4^heart ^mice is diminished along the entire rostral to caudal axis, with a drastic decrease in density and near absence of INs in posterior cortex (Figure 8). In WT P0 mice, Reelin-expressing INs are preferentially located in anterior CP (Figure 8D). In the ErbB4-/- HER4^heart ^mice, the presence of Reelin-expressing cells within the CP is significantly reduced, while the presence of Reelin-expressing cells in the MZ, indicative of Reelin-positive Cajal-Retzius neurons that are distinct from ErbB4-expressing, MGE-derived INs, remains relatively normal (Figure 8D'). In conclusion, these results are consistent with our findings presented in the preceding sections that NRG-ErbB4 signaling plays an important role in directing the tangential migration of GABAergic INs through the vTel and to their cortical destinations.

## Discussion

The migration of INs from MGE to cortex is controlled by a complex combination of long-range and short-range attractant and repellent signals, as well as cell-adhesion complexes and motogenic factors [12]. Most GABAergic INs that populate the cortex are generated in the MGE, take discrete migratory pathways through the vTel to the cortex and within the cortex, and express the receptor tyrosine kinase ErbB4 during their migration [28,29].

During their migratory path, INs generated in the MGE traverse the vTel, which includes the nascent striatum and other components of the basal ganglia. It was shown previously that the migrating INs are directed by Sema3A and 3F, two chemorepellents expressed by the developing striatum [13]. In this study, we have used *in vivo *and *in vitro *analyses and experimental manipulations of WT and ErbB4 mutant mice to study the roles for NRG-ErbB4 signaling in regulating the migration of INs from the MGE to their cortical destinations. We demonstrate that two Nrg1 isoforms, Nrg1-type I and Nrg1-type III, as well as Nrg3, function as repellents for migrating ErbB4-expressing INs and create barriers that help define their migratory pathways and appear to funnel them from the MGE and through the vTel to cortex. Similar to the chemorepellents Sema3A and 3F expressed in the nascent striatum [13], the expression domains of NRGs show minimum overlap with ErbB4-expressing INs. Moreover, the expression pattern of Nrg1-type III (Figures 1, 2, and 3) is very similar to the expression pattern previously reported for Sema3A [13]. Thus, it is likely that NRGs, together with Sema3A and 3F, delineate through a repellent mechanism barriers to IN migration, and these barriers form corridors of low repellent expression upon which MGE-derived INs are concentrated; these corridors appear to funnel the migrating INs through the vTel into cortex.

We have carried out five distinct types of experiments and analyses: 1) extensive expression analyses of NRGs relative to the distributions of ErbB4-expressing INs and their migratory paths from the MGE to the cortex; 2) changes in the distributions of ErbB4-expressing INs relative to NRG expression domains when NRG-ErbB4 signaling is eliminated in ErbB4-deficient mice; 3) collagen co-culture assays of the influences of secreted NRGs on migrating MGE cells; 4) migration assays using living forebrain slices to determine the migration patterns of WT and ErbB4-deficient MGE cells relative to domains of endogenous NRG expression; and 5) *in utero *electroporation experiments to determine the influences of ectopic NRG expression domains within the vTel migratory paths on the migration and distribution of ErbB4-expressing, MGE-derived INs. The findings from each of these studies are consistent with one another and support a repellent or inhibitory function for NRG-ErbB4 signaling on IN migration.

A function for Nrg1 on directing the migration of ErbB4-expressing, MGE-derived INs has been previously reported by Flames *et al. *[23], but our findings fundamentally differ from theirs. Whereas we demonstrate that Nrg1 isoforms and Nrg3 act through ErbB4 to inhibit or repel migrating MGE-derived INs, Flames *et al. *[23] concluded that the membrane-attached isoform of Nrg1 (Nrg1-type III; referred to as Nrg1-CRD by Flames *et al. *[23]) provides a growth permissive corridor through the developing striatum and that the secreted Ig isoform of Nrg1 (Nrg1-type I; referred to as Nrg1-Ig by Flames *et al. *[23]) attracts INs from the vTel into the cortex and along the IZ.

If NRGs do influence the migration of cortical INs via ErbB4, then a straightforward assessment of whether NRGs act as repellents or attractants for ErbB4-expressing INs would be indicated by the relationship of their expression patterns relative to one another in WT mice and changes in these relationships in ErbB4 mutant mice. If the expression patterns of NRGs and the distributions of ErbB4-expressing INs complement one another in WT mice, NRGs likely act as repellents and are very unlikely to act as attractants, whereas a finding of significant overlap between NRG expression and ErbB4 IN distributions would suggest that the NRGs may act as attractants. At each embryonic age that we examined, ErbB4-expressing INs are clearly distributed in patterns that avoid domains of NRG expression. At each age (Figures 1, 2, 3, and 4), we find that high densities of ErbB4-expressing INs are present where NRG expression is low or non-detectable, and, conversely, a low density of ErbB4-expressing INs is found where significant NRG expression is detected. These complementary patterns of NRG expression and the distributions of ErbB4-expressing INs are observed both in the vTel and within the cortex, with the patterned distribution of INs often abutting domains of NRG expression, or sculpted to fill in 'holes' or channels in the NRG expression pattern. Within the vTel, the INs are funneled through these channels to the cortex, and within the cortex, ErbB4-expressing INs tangentially migrate selectively within the MZ and IZ where NRG expression is low, appearing to avoid the Nrg3 expression domain in the CP and the Nrg1 expression domain in the VZ/SVZ. These expression data are straightforward, and fit the prediction for a repellent function of the NRGs and are inconsistent with an attractant function. Based on these expression data in WT mice, therefore, one must conclude that if these NRGs do indeed influence IN migration, that they act as repellents and not as attractants. We do find some overlap of ErbB4-expressing INs with NRG expression domains, but this can be explained by the certainty that the INs are influenced to varying degrees by both positive and negative guidance cues, and that as the INs migrate they not only encounter a complex mix of signals, both repellents and attractants, but likely also have differential expression of receptors for these signals. Therefore, the migration of the INs is controlled by a combinatorial mix of signals and responses, with some being dominant over others, and the overall balance varying in both a time- and place-dependent fashion.

NRGs as repellents for tangentially migrating INs is strongly supported by our findings from two additional sets of experiments analyzing differences in the responses of migrating MGE-derived INs with or without NRG-ErbB4 signaling intact, including: 1) changes in the distributions of MGE-derived INs relative to NRG expression domains in ErbB4-deficient mice (ErbB4-/- HER4^heart ^mice) compared to their WT littermates; and 2) differences in the distributions of MGE cells relative to NRG domains as they migrate from MGE explants dissected from WT or ErbB4-deficient mice placed on living WT forebrain slices.

In the first set of experiments, we find that the complementary patterns of the distribution of ErbB4-expressing INs and domains of NRG expression are substantially degraded in mice with a targeted deletion of ErbB4 (ErbB4-/- HER4^heart ^mice). The complementary patterns are not completely lost, likely because of other persisting activities, such as semaphorins shown to act as repellents for INs [13]. When NRG-ErbB4 signaling is eliminated in the MGE-derived INs, their distribution broadens and a substantial proportion move into domains of NRG expression, again consistent with a repellent function for the NRGs in WT mice. To explain these findings in the ErbB4 mutant by an attractant mechanism would require that the distribution of INs is focused on domains of NRG expression in WT, and following the loss of NRG-ErbB4 signaling, their distribution broadens as INs move away from domains of NRG expression and into locations where NRGs exhibit low expression. Again, this is the opposite of what we find, as our expression analyses of WT mice show that ErbB4-expressing INs are not focused on NRG expression domains but are concentrated in regions of low or non-detectable NRG expression, being focused on migratory paths that abut domains of NRG expression, or are hemmed by them. In other words, our findings show that rather than NRG expression defining through an attractant mechanism a permissive migration path upon which ErbB4-expressing, MGE-derived INs are normally focused, the IN migration paths are defined as corridors of low NRG expression present within the NRG expression domains, and the INs are focused in these channels by a repellent influence of NRGs.

A second set of experimental findings that strongly supports the repellent function of NRGs for MGE-derived INs is the migration patterns of WT and ErbB4-deficient MGE cells relative to domains of endogenous NRG expression in living forebrain slices from WT mice. In Figure 6, we demonstrate that cells migrating from WT MGE explants show a tendency to avoid entering the NRG expression domains, which is evident for the distribution of MGE cells relative to the expression domains of Nrg1-type III in the nascent striatum and Nrg3 in the CP (Figure 6). For example, WT MGE cells form an abrupt wall at the borders of domains expressing Nrg1-type III and Nrg3 and are largely excluded from these domains. We find that this strongly patterned migration is not exhibited by cells migrating out of MGE explants derived from ErbB4-/- HER4^heart ^mice and instead these ErbB4-deficient MGE cells are distributed in a radial pattern with no apparent response to the NRG expression domains. These results demonstrate that the patterned distribution of WT MGE cells and their avoidance of NRG expression domains is due to an ErbB4-mediated repellent response of MGE cells to endogenous NRGs expressed in the forebrain slices. Further, these findings indicate that the defects in the migration of MGE-derived INs resulting from the targeted deletion of ErbB4 is cell-autonomous to the MGE-derived INs themselves, rather than due to secondary defects resulting from the ErbB4 deficiency.

The experimental findings described above address the influences of endogenous NRGs and the responses of MGE-derived INs to them within essentially an *in vivo *setting. These types of experiments are distinct from reduced experimental scenarios such as transfection-based experiments using collagen co-cultures or membrane carpet assays, and complement them well. In summary, the expression analyses of WT and ErbB4 mutants, the migration assays using WT and ErbB4-deficient MGE explanted onto living forebrain slices, and the results from the collagen gel co-culture migration assays together demonstrate that NRGs are repellents for ErbB4-expressing, MGE-derived INs.

Another set of experiments that support our interpretation that NRGs act as repellents for migrating INs comes from our use of *in utero *electroporations to ectopically express NRG isoforms within the migration path of MGE-derived INs. If NRGs were attractants for the migrating ErbB4-expressing cortical INs, we would expect to observe that INs would either pass through the ectopic NRG expression domain, consistent with the function of defining a permissive corridor *per se*, or possibly accumulate within it, similar to the accumulation of INs in ectopic expression domains of the chemoattractant Cxcl12/SDF1 [18,32]. In contrast, if NRGs act as a repellent or inhibitor for the migrating INs, we would expect that the INs would not enter the ectopic domains of NRG expression and would accumulate outside of them or deviate away from them. Our findings are in agreement with the latter prediction: most ErbB4-expressing INs do not enter an ectopic NRG expression domain within their vTel migratory path and accumulate proximal to the electroporation site. Further, this migration blockade of MGE-derived INs results in a substantial decrease of ErbB4-expressing INs within the cortex distal to the electroporation site, a so-called 'shadow' effect. Taken together, these findings strongly argue that NRGs act as repellents for migrating ErbB4-expressing, MGE-derived INs and that their expression domains serve as barriers for the migration of ErbB4-expressing INs to funnel them from the MGE to the cortex.

We find that diminished numbers of INs reach their final destination in the cortex in the ErbB4-/- HER4^heart ^mice, a result that we agree upon with Flames *et al. *[23]. Interestingly, although we fundamentally differ on the underlying mechanism, that is, diminished NRG-ErbB4 mediated repulsion versus attraction, as we discuss above, both scenarios would result in the defective migration of MGE-derived INs due, at least in part, to a failure of the INs to be properly focused on their migratory path. Our findings suggest that the diminished numbers of INs in the ErbB4 mutant cortex is due to a failure of migrating INs to be properly focused upon the corridors within the vTel that normally funnel them through vTel and into the cortex, resulting in them being aberrantly scattered within the vTel.

Within the cortex, the migration of ErbB4-expressing INs is dynamic: they first migrate tangentially in the MZ and IZ/SVZ, then switch to take a radial migratory path to reach their final laminar location [37]. During the tangential migration phase, NRG expression is detected in the CP and VZ/SVZ, in a complementary pattern to the distribution of the migrating ErbB4-expressing INs. Later in development, however, INs do invade the CP and many studies have suggested that the process of CP invasion by GABAergic INs is temporally regulated. It is likely that this change from a tangential to radial migration is due to both INs changing their responsiveness to repellent signals expressed in the CP as well as the level of expression of these repellents. Using stripe assays, it has been shown that the CP undergoes an age-dependent maturation during which an initially repellent influence becomes strongly diminished [38]. Consistent with this observation, at later developmental stages, NRG expression is downregulated in the CP (unpublished observations), although its expression is retained in a subset of adult cortical neurons [39]. In addition, INs respond differently to signals within their migratory paths and the CP during their tangential and radial migration periods [18,19]. For example, INs migrate radially away from the expression domains of the attractant Cxcl12 in their tangential migratory paths in the MZ and IZ/SVZ to enter the CP, even though Cxcl12 expression is maintained in the MZ and IZ/SVZ during this period [19].

In conclusion, we demonstrate a novel role for NRGs (Nrg1-type I, Nrg1-type III, and Nrg3) acting as repellents signaling through the receptor tyrosine kinase ErbB4 to control the tangential migration of GABAergic INs from the MGE to their cortical destinations. In addition to roles in controlling the migration of GABAergic INs, other studies have shown roles for ErbB4 signaling later in cortical development - for example, in influencing the development of inhibitory cortical circuits [39]. These and other studies are beginning to reveal significant defects in neural structure and function resulting from a compromised NRG signaling pathway and may begin to provide insight into the potential relationship between ErbB-NRG signaling and schizophrenia, initially based upon the identification of Nrg1 and ErbB4 as susceptibility genes in schizophrenia [40-46]. Further studies on the functions of NRG/ErbB signaling during brain development may provide us with a better understanding of these and related neurological disorders.

## Materials and methods

### Mice

ErbB4+/+ HER4^heart^, ErbB4+/- HER4^heart ^and ErbB4-/- HER4^heart ^mice were generated and genotyped by PCR as described [30]. For *in vitro *transplantation assays and explant cultures the heart-rescued ErbB4 knockout mice were bred with GFP-expressing transgenic mice [31]. ICR outbred mice (Charles River Laboratories, Wilmington, MA, USA) were used for *in utero *electroporation experiments. Midday of the day of vaginal plug detection was considered E0.5, and the day of birth is termed P0. All research and procedures carried out on mice in this study conform to NIH guidelines and have been approved by our institution's animal care and use committee.

### *In situ *hybridization

For *in situ *hybridization on sections, brains were fixed with 4% paraformaldehyde in PBS, cryoprotected with 30% sucrose in 0.1 M PBS, embedded in Tissue Tek OCT compound (Sakura Finetek, Torrance, CA, USA) and cut at 14 to 20 μm on a cryostat. *In situ *hybridization using ^35^S-labeled riboprobes and counterstaining with DAPI were performed as described previously [47]. The ErbB4 probe spans sequence #471-1262 of the mouse ErbB4 cDNA in NCBI Reference Sequence NM_010154.1.

### *In vitro *assays

For *in vitro *explant cultures: the EGF domains of mouse Nrg1α, Nrg1β and Nrg3 were subcloned into pSecTagB (Life Technologies, Carlsbad, CA, USA) containing the Ig κ-chain leader sequence that facilitates secretion. The full-length Nrg1-type I, -type II and -type III were cloned into pcDNA vector. 293T cell were transfected with PolyFect Transfection Reagent (QIAGEN, Valencia, CA, USA). The transfected 293T cells were aggregated by centrifugation and immobilized with collagen/matrigel (1:1) using rat tail collagen gel (Beckton-Dickinson, Franklin Lakes, NJ, USA). The brains from E14.5 mice were dissected out, and coronal sections of 300 μm made with a Brinkmann tissue chopper (Labequip, Markham, Ontario, Canada). Then the SVZ of the MGE was isolated and trimmed into blocks of 300 μm. The trimmed cell aggregates and MGE explants were embedded in collagen/matrigel (1:1). The distance between the cell aggregates and the explants was 100 to 200 μm. Culture medium was 10% fetal calf serum, 100 μg/ml of penicillin and streptomycin in D-MEM/F12 (Cellgro, Manassas, VI, USA); culture conditions were 37°C, 5% CO_2 _for 24 to 48 hours. The distributions and directional movement of cells migrating away from the explants were scored by analysis of fluorescence labeling using Image J software as described in the legend for Figure 5 and in Pak *et al. *[48]. For *in vitro *transplantation and slice culture, MGE explants and host slices were obtained at E14.5. MGE explants were placed on the host slices and cultured for 48 hours.

### *In utero *electroporation

Expression constructs were made by subcloning the EGF domains of mouse Nrg1α, Nrg1β and Nrg3 into a Bluescript SK vector containing the cytomegalovirus (CMV) early enhancer element and chicken β-actin promoter and a polyA sequence of bovine growth hormone sequences (pCAG vector). E12.5 embryos were visualized through uterus with a fiber optic light source. DNA solutions containing 0.5 μg/μl pCAG-NRGs + 0.2 μg/μl pCAG-eGFP + 1% fast green (Sigma, St Louis, MO, USA) were injected with a glass capillary into the left ventricle of each embryo and electroporated with Paddle-type electrodes (CUY21 Electroporator: Nepa Gene, Ichikawa, Chiba, Japan) in a series of five square-wave current pulses (35 V, 100 ms × 5). The electroporated embryos were allowed to develop until E17.5 and selected for further analyses by direct visualization of eGFP expression. The survival rate of embryos was approximately 80%. The distributions of ErbB4-expressing cells relative to the transfection domains were visualized by *in situ *hybridization with the ErbB4 probe.

## Abbreviations

CGE: caudal ganglionic eminence; CP: cortical plate; CRD: cystein rich domain; dTel: dorsal telencephalon; E: embryonic day; EGF: epidermal growth factor; eGFP: enhanced green fluorescent protein; EGFR: epidermal growth factor receptor; GABA: γ-amino butyric acid; GE: ganglionic eminence; IN: interneuron; IZ: intermediate zone; LGE: lateral ganglionic eminence; MGE: medial ganglionic eminence; MZ: marginal zone; NRG: neuregulin; P: postnatal day; SVZ: subventricular zone; vTel: ventral telencephalon; VZ: ventricular zone; WT: wild type.

## Competing interests

The authors declare that they have no competing interests.

## Authors' contributions

All authors read and approved the final manuscript. Specific contributions are as follows: HL and S-JC designed and performed the primary experiments, prepared figures and assisted in writing the paper. TH and CGPG helped perform parts of the study and writing the paper. DO'L conceived and designed the study, helped analyze material and prepare figures, and wrote the paper.
